# Isolation of high-purity myenteric plexus from adult human and mouse gastrointestinal tract

**DOI:** 10.1038/srep09226

**Published:** 2015-03-20

**Authors:** David Grundmann, Markus Klotz, Holger Rabe, Matthias Glanemann, Karl-Herbert Schäfer

**Affiliations:** 1 ENS Group, University of Applied Sciences Kaiserslautern/Zweibrücken, Germany; 2 Department of General, Visceral, Vascular and Pediatric Surgery, Medical Faculty of the University of Saarland, Homburg/Saar, Germany; 3 University of Heidelberg, Paediatric Surgery Mannheim, Germany

## Abstract

The enteric nervous system (ENS) orchestrates a broad range of important gastrointestinal functions such as intestinal motility and gastric secretion. The ENS can be affected by environmental factors, diet and disease. Changes due to these alterations are often hard to evaluate in detail when whole gut samples are used. Analyses based on pure ENS tissue can more effectively reflect the ongoing changes during pathological processes. Here, we present an optimized approach for the isolation of pure myenteric plexus (MP) from adult mouse and human. To do so, muscle tissue was individually digested with a purified collagenase. After incubation and a gentle mechanical disruption step, MP networks could be collected with anatomical integrity. These tissues could be stored and used either for immediate genomic, proteomic or in vitro approaches, and enteric neurospheres could be generated and differentiated. In a pilot experiment, the influence of bacterial lipopolysaccharide on human MP was analyzed using 2-dimensional gel electrophoresis. The method also allows investigation of factors that are secreted by myenteric tissue in vitro. The isolation of pure MP in large amounts allows new analytical approaches that can provide a new perspective in evaluating changes of the ENS in experimental models, human disease and aging.

The ENS is responsible for the regulation of homeostatic key features in the gastrointestinal tract, such as gastric secretion, absorption and motility^1^,^2^. It consists of several intricate neural networks within the gut wall, which harbors up to several hundred million neurons and enteric glial cells, as well as neuronal progenitor cells, throughout its lifetime^3^,^4^,^5^. These progenitor cells enable the ENS to respond to changes of the inner and outer environment, dietary habits and diseases. The ENS is not only involved in gastrointestinal diseases such as inflammatory bowel diseases and enteric neuropathies; there is increasing evidence that the ENS is affected in systemic disorders such as diabetes and neurodegeneration^6^,^7^,^8^,^9^,^10^,^11^,^12^,^13^,^14^,^15^. This is why the ENS has been increasingly studied by scientists and clinicians of various disciplines during the last two decades and has ranged from both an important part of the problem or disease to a suitable and realistic autologous neural stem cell source for the treatment of peripheral and central nervous system impairments^16^,^17^,^18^.

Enteric neurons are very well classified by their anatomical localization, morphology, marker expression and functional properties. Enteric neurons play a pivotal role in inflammatory bowel disease pathogenesis. Changes in neuronal numbers can influence the impact of inflammation in different models of inflammatory bowel disease^19^. Moreover, adult neurogenesis occurs in the mouse intestine in vivo in response to 5-Hydroxytriptamine 4 (5-HT4) receptor activation or intestinal disruption and injury when glial cells transdifferentiate into neurons^20^,^21^,^22^. Although the number of myenteric neurons in mice is not affected during the undisturbed aging process, it seems to be decreased in a transgenic mouse model simulating Alzheimer's disease^13^,^23^.

Similar to neurons, enteric glial cells consist of several subpopulations that can be discriminated by their anatomical localization, morphologies or marker proteins such as GFAP, S100b, etc. Their divergent functional properties have recently begun to be examined in detail^3^. Nevertheless, it is already known that enteric glial cells can modulate gut inflammation and influence intestinal homeostasis, as has been demonstrated by conditional ablation of glial cells. This ablation results in the disruption of the epithelial barrier and consecutive intestinal inflammation^24^,^25^. As recently shown in the adult mouse model, enteric glia cells can also proliferate during both steady state and after intestinal injury in vivo^20^,^21^.

Although the ENS works autonomously, it is strongly connected to the central nervous system by sympathetic and parasympathetic (vagal nerve) fiber systems, the so-called “Brain-Gut-Axis”^26^. This connection allows a mutual exchange of information, and might be responsible for spreading viruses, prions or pathological peptides from the gut to the brain. Thus, there is evidence that diseases of the brain, such as Parkinson's disease or developmental disorders in the central nervous system, might originate in the ENS in response to environmental toxins, viral infections or alterations in the microbiome^27^,^28^,^29^.

Taken together, the ENS exerts a strong functional influence on the physiology and pathophysiology of the intestine and other organ systems. Despite the increasing knowledge of the influence of the ENS, further studies are necessary to address specific questions on a molecular level. Protein composition changes, signaling pathway alterations or soluble factor secretion by the ENS under different conditions remain unclear for the ENS while it is embedded in the gut wall. Here we do see limitations in most of the techniques applied to date. The isolation of pure adult myenteric tissue in transgenic mouse models and healthy, as well as diseased, human patients, allows investigation of alterations within the ENS on a molecular level and provides the perspective of comparing mouse and human data collected from the same pure tissues. This provides new perspectives for translating findings from mouse models to humans. In this study, we established a reproducible method by which the MP can be isolated with anatomical integrity from all gastrointestinal segments of the adult mouse (stomach, duodenum, jejunum, ileum, caecum, proximal and distal colon), as well as from the stomach and colon in human gut tissue with a very high purity.

## Results

To isolate MP from adult tissue, a new algorithm for the isolation procedure was developed. MP was isolated from the smooth muscle, which had to be separated manually from the submucous layer prior to digestion, using watchmaker forceps. Although the tissues from the individual gut segments vary significantly, the muscle layer can be removed in all segments of the gastrointestinal tract (GIT). The individual segments present large variation in their ease of separation. Although stomach and caecum are most difficult to dissect, small intestinal and colonic segments are much easier and do not provide major obstacles, such as muscle layer thickness or strong interconnections to the submucosal layer, which often lead to muscle layer ruptures.

**Figure 1:**
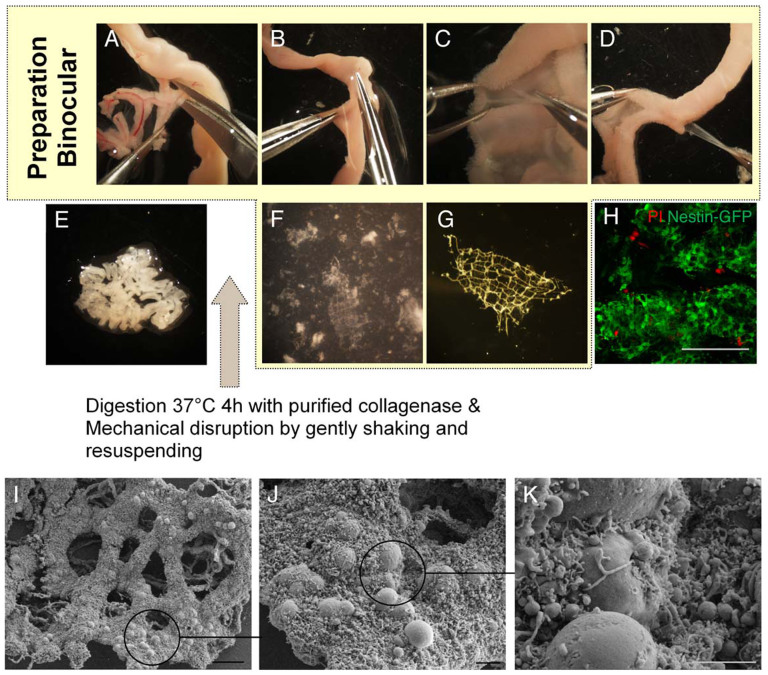
Isolation procedure for myenteric plexus from adult mouse after dissection of the gastrointestinal tract: Removal of the mesentery (A), opening of the intestinal segments along the mesenterial line (B), dissecting the muscle from the mucous layer (C) and pulling the muscle sheath without rupturing it (D). The isolated muscle layer was cut into small pieces (E). After the digestion and first mechanical disruption step, the plexus pieces are not yet clearly visible (F, 30x) and have to be further purified (G, 50 x). Propidium iodide (PI) incubation shows only single dead cells in a Nestin-GFP-mouse derived myenteric plexus (H). Magnification 200× scale bar 100 μm. (I–K) Scanning electron microscopical images demonstrate the purity and removal of adjacent cells from the myenteric plexus of the proximal colon. Scale bars (I) 50 μm, (J) 10 μm, (K) 5 μm.

Adult (Nestin-GFP) mice were used in these experiments to demonstrate not only the feasibility of the isolation procedure in all segments but also the abundance of intrinsic neural stem cells that can easily be cultured (Fig. 1 A–G).

In former studies, crude enzyme cocktails (CLSII) were used to isolate MP from postnatal tissue. The enzyme was used at a concentration of 1 mg/ml, which yielded sufficient amounts of MP to perform tissue culture experiments. In adult mice and humans, this method delivered only single ganglia in very low numbers^32^, which were enough to perform single tissue culture experiments but were not sufficient to perform proteomic approaches or liberation studies. We compared the yield of the formerly used CLSII at an increased concentration of 10 mg/ml with a highly purified collagenase, Liberase TH (Roche). The Liberase could be used at a concentration of 0,75 mg/ml. In the Liberase approach, hundreds of large networks can be harvested from one individual small or large intestine, whereas the CLSII digestion at a low concentration (1 mg/ml) does not allow any network collection. Single fragments of networks could be harvested only when higher concentrations of CLSII were used (20 mg/ml) (supplementary figure 1). These high concentrations are critical because of the contamination with high amounts of additional enzymes, such as trypsin or other proteinases. These can be rather problematic, especially in cases of longer digestion periods. Liberase TH allows an extended incubation time without over-digestion of the target tissues used. The ENS does not contain collagen; thus, Liberase can easily be used for overnight digestions, e.g., in human material.

Another important factor concerning analysis of isolated tissues is the purity. A huge advantage of the described method is the removal of the surrounding cells. Using scanning electron microscopy (SEM), we could clearly demonstrate that smooth muscle cells and connective tissue are completely removed after the digestion procedure (Fig. 1 SEM pictures). Scanning electron microscopical pictures of the plexus surfaces show only neuronal tissue. Moreover, western blot analysis of isolated MP did not show smooth muscle actin. The procedure is very gentle and avoids excessive cell death, as demonstrated by live-dead assays (Fig. 1H) and the absence of apoptotic blebbing. The latter could be proven using scanning electron microscopy (Fig. 1 I–K).

**Figure 2:**
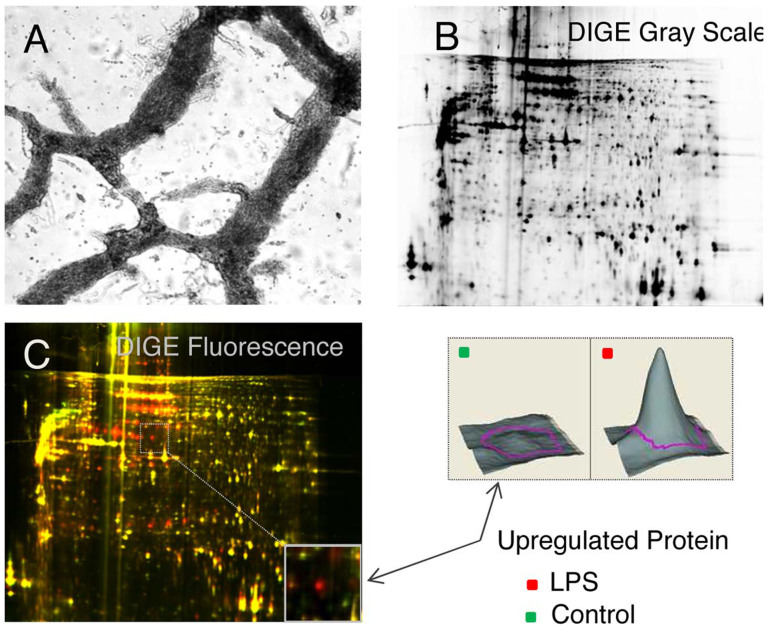
(A) Proteomic analysis of human stomach myenteric plexus after endotoxin stimulation. Myenteric Plexus networks were isolated with purified collagenase and incubated with and without 5 μg/ml LPS for 24 h. (B) The protein expression was analyzed using DIGE. The gray scale image shows a high quality of the 2D-gel. (C) The protein expression patterns (merge, yellow) from LPS stimulated (red) and unstimulated (green) samples show differentially expressed spots, which demonstrate mainly protein upregulation under LPS treatment.

With the application of Liberase, complete networks instead of single ganglia can be harvested in much larger quantities This is especially critical for proteomic approaches, where huge amounts of protein are needed (Fig. 2). Moreover, by obtaining complete networks, the cells within the interconnection strands can also be harvested. These harbor glial and neural stem cells that otherwise would be lost completely.

The incubation time has to be adapted slightly to the individual gut segments. Approximately 4 hours (+/−30 minutes) is sufficient to isolate the major part of MP from the muscle layer. Although the digestion time of duodenum and caecum can be reduced (3.5 h), it has to be extended in proximal and distal colon (4.5 h). The prolonged digestion did not affect the anatomical integrity of the MP.

**Figure 3:**
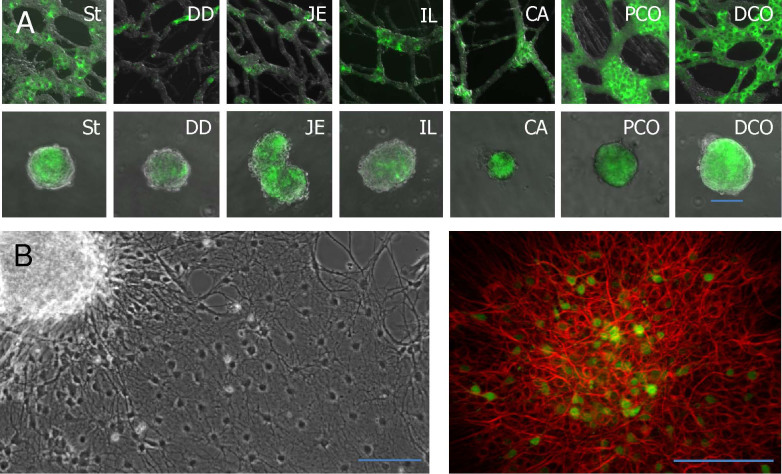
(A) Isolated myenteric plexus with neurospheres from corresponding segments of adult Nestin-GFP (green) mouse: St = stomach, DD = duodenum, JE = jejunum, IL = ileum, CA = caecum, PCO = proximal colon, DCO = distal colon. Magnification 200×. Scale bar 100 μm. (B) A differentiated neurosphere from adult mouse colon and a differentiated neurosphere from Nestin-GFP (green) embryonic mouse stained against ßIIItubulin (red). Magnification 100×, as well as 200×. Scale bar 100 μm.

Although the isolation procedure with Liberase is much easier to perform and gentler than crude collagenases, there are single segments that can be more problematic. Stomach and caecum tissues should be handled more carefully. In these parts of the GIT, the structure of the MP is more delicate than in other segments (Fig. 3A). In the caecum, the myenteric ganglia are more isolated and the interconnecting strands are weaker. In the stomach, the connections between the cells are even weaker and the ganglia can disaggregate when aspirated in the pipette tip during the collection process.

The presented method also represents a basic approach that can be used in a modified manner for the isolation of MP from other developmental stages of rodents, pig or human. The incubation time has to be optimized in younger animals (2.5 hours postnatal mouse stage P0-P3, 1.5 hours embryonic mouse stage E18), and additional mechanical disruption steps during incubation have to be applied in adult rats because of more compact muscle layers (gently resuspending 2 times each hour). Adult human smooth muscle tissue can be treated likewise with a modified approach. Stomach tissue was chosen as the most difficult tissue for the isolation process. Here, the tissue has to be incubated overnight and the Liberase diluted in tissue culture medium (DMEM/F12, Invitrogen). The amount and quality of the isolated plexus is similar to that of the mouse tissues (Fig. 2A).

After isolation, the MP-networks can be used immediately for experiments on the proteomic or transcriptional level depending on the part of the GIT. There is sufficient RNA or protein available in case the whole gut is digested. Thus, it is possible to isolate 1 μg RNA per large intestine from adult mice, which is sufficient to perform PCR or gene array experiments. The protein yield per small or large intestine from one mouse ranges from 20 to 30 μg. In human samples, depending on the size of the tissue, several hundred μg can be obtained.

As a proof of principle for inflammation, MP was collected from human stomach, stimulated with LPS overnight and analyzed using 2D-DIGE (Fi. 2 B and C). A total of 2082 individual proteins could be identified, from which 131 were upregulated (113 proteins) and 18 were down-regulated (18 proteins) in the LPS treated condition. Because of the primary purity of the tissues used, the quality of the DIGE results was very high and the variation between the individual experiments very low.

**Figure 4:**
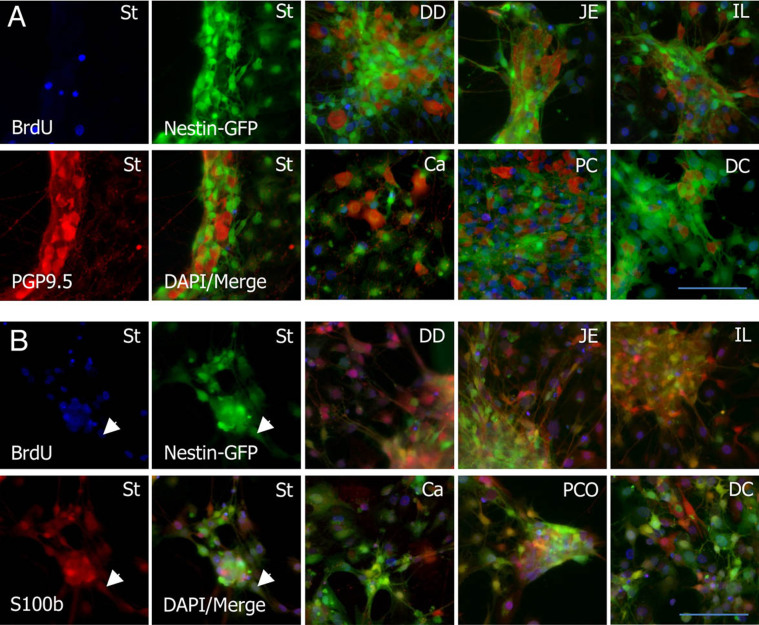
Immunofluorescence stainings of neurospheres differentiated on ECM-coated coverslips for 6 days. Proliferation was revealed using BrdU-incorporation (blue). The phenotype of the cells was visualized via the expression of Nestin (green) and the pan-neuronal marker PGP9.5 (red) for neurons (A) and S100b for glial cells (B). The proliferation was detected in the S100b+ nestin-expressing glial population. Individual stainings are depicted for the stomach (St) only. All other segments are shown as merged images. Magnification 200×. Scale bar: 100 μm.

The isolated networks can be used for cell culture experiments or to isolate neural stem cells in neurosphere approaches from all segments of the GIT. To generate neurospheres, the MP networks were used without dissociation and neurospheres did arise within 3–6 days (Fig. 3A). The neurospheres could easily be differentiated to neurons or glial cells when grown on extracellular matrix coated coverslips (Fig. 3B). Cell proliferation could be observed in S100b+ glial cells only. PGP 9.5+ neurons did not show any sign of proliferation under these culture conditions as revealed by the BrdU incorporation (Fig. 4).

**Figure 5:**
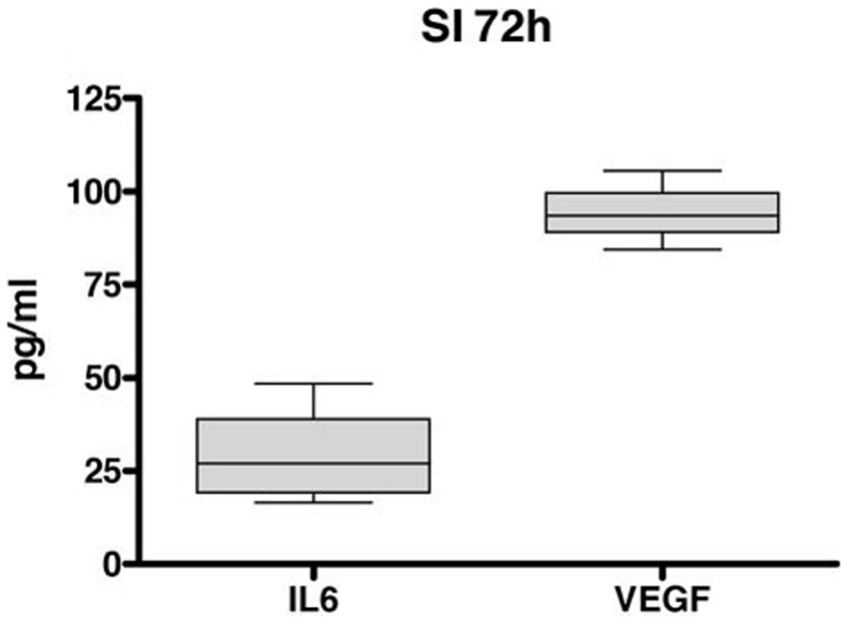
Multiplex-ELISA data from isolated myenteric plexus: Release of soluble factors from the myenteric plexus culture after 72 h of the small intestine of adult mouse. The supernatant was collected and analyzed using multiplex assay (Luminex). A release of IL-6 and VEGF was detected after 72 h. n = 5 SI small intestine.

The described method also allows investigation of the auto- or paracrine activity of the cells within the MP by culturing intact MP networks for a certain period of time. After this period, the supernatant can be collected and analyzed using multiplex ELISAs for different cytokines. The high amount of isolated MP per segment allows investigation of, e.g., cytokine release or stimulation with spatial resolution. This is especially interesting when looking at incretins that might have segmental distinct influences. As a proof of principle, we cultured small intestinal MP without any stimulation. The supernatants were collected after 72 hours and were immediately analyzed. A broad range of cytokines could be identified in varying extents. Although MCSF, G-CSF, IL10, LIF, TNFα and IL17 were detected at only very low levels, IL6 and VEGF could be found in significant amounts (Fig. 5).

In principle, the freshly isolated or cultivated myenteric tissue can be used for all kinds of analytical or functional experiments. Calcium imaging and multi electrode array experiments were added as supplements to prove the feasibility (Supplemented figure 2 and 3).

## Discussion

To date, there is no cell surface marker available to perform cell sorting techniques and to isolate all ENS-cells in one step, as can be done with leukocytes^30^ using CD45 antibodies. Here, the isolation of intact MP provides an opportunity to investigate the ENS with a purity and integrity that cannot be achieved by other methods. The enzymatic isolation of the MP has already been established and reported for the gut of fetal, postnatal and adult human intestine and also of postnatal rat. These methods are laborious, time consuming and not applicable for untrained operators or operators with minimal experience in primary tissue handling. In adult tissue, including rat, mouse or human, only single or a few interconnecting ganglia could be harvested by a combination of enzymatic digestion and manual dissection^31^,^32^,^33^. Although RT-PCR techniques allow investigation of regulated mRNA, investigation of the translated proteins affords a relatively large amount of whole enteric neural tissue. Additional experiments for cytokine release or dissociated cultures were not possible. The small amount of tissue does not deliver a sufficient release of cytokines. Proteomic approaches have been far from realistic. To perform single 2D-gel electrophoresis, a large number of newborn animals have to be used. The protein yield of one single postnatal animal is in the range of a few μg, which is not enough to perform more sophisticated proteomic approaches. To obtain a sufficient amount of protein (30–50 μg for analytical and 250 to 500 μg for preparative 2D gels), a large number of animals have to be dissected^34^. The new approach allows isolation of sufficient quantities of protein to perform individual and even spatial analysis of MP from different gut segments. Independent of low tissue yields, the newborn and embryonic tissues have a severe disadvantage because they do not represent the situation in the adult gut. Because most diseases that are of interest in gastrointestinal research or that potentially use the ENS as a diagnostic marker, e.g., neurodegeneration, are within the adult range, we focused on the isolation of adult MP-tissue. Adult MP could already be isolated by us or other groups but, to date, not in these amounts or with this level of purity. Several groups could obtain adult MP to perform in vitro experiments, electrophysiological studies or transplantations^32^,^35^,^36^,^37^; however, the MP used either had cellular contamination (smooth muscle) or was obtained in only low amounts. Usually, tissue was isolated from only one region.

In the present study, Liberase was used, consisting of purified Collagenase I and II. Liberase also contains other proteases (e.g., thermolysin), but these are far less harmful than the ones in crude collagenase preparations. The latter contain aggressive digestive proteinases, such as trypsin or caseinase^33^.

The digestion of the tissue with these specific enzymes leads to a much larger amount of isolated MP-networks than classical collagenase, especially in adult tissues (supplemented Fig. 1).

Using crude collagenase for the isolation of the MP from adult mouse, we collected only some ganglia and a very small amount of MP-networks, which were not enough to perform, e.g., proteomic experiments compared to the isolation with Liberase. In the stomach, where the muscle is thick and the MP-networks are fragile, MP-networks or ganglia could not be isolated at all when crude collagenase was used.

The specific enzyme cocktail gives the advantages of an extended incubation time of several hours, which differs from crude collagenases and does not interfere with the integrity of the isolated tissue. Over time, digestion with crude collagenases leads to complete digestion of all tissues, mainly by the additional proteases. Because of the fact that there is no collagen within the MP, the enzyme digests the surrounding tissues without affecting the ENS. This allows the use of the tissue for experiments where the cells have to be vital, such as releasing experiments or cell culture.

In addition, the activation of the cells by endotoxin should be minimized because the endotoxin level is several thousand-fold reduced in purified collagenase during manufacturing. These purified enzymes are also available on GMP status if the cells will be used for transplantation into living hosts. Thus, the risk of immune modulation or in vitro co-stimulation of the cells as a result of enzyme cocktail contamination is extremely reduced.

Referring to all these advantages, we can achieve high consistency between the individual preparations and even between different laboratories. A potential contamination, by e.g., smooth muscle cells, can be avoided.

We are using the isolated MP, e.g., to investigate proteomic changes in health and disease. Based on the purity and amount of tissue, it is possible to investigate changes within developmental stages (embryonic-postnatal-adult), spatially distinct sections or between healthy and diseased tissue. Thus, this method is a perfect tool for comparing alterations of the ENS in transgenic or disease mouse models as well as human tissue from diseased patients. This is particularly interesting in neurodegenerative or inflammatory diseases, where early changes of the ENS might be used as predictive biomarkers^13^. For this kind of experiments, it is an enormous advantage to have very consistent individual experiments.

As an example for an experimental approach, we have shown that the proinflammatory endotoxin LPS, a molecule found in the outer membrane of Gram-negative bacteria, can highly affect the human ENS by changing its protein composition easily. Moreover, the use of transgenic animals, where i.e. neural stem cells (GFP-Nestin), neurons or glial cells are expressing fluorescent dyes, allows to identify and sort specific cellular populations. Proteomic (or genomic) changes within only neurons or glial can thus easily be identified.

The adult mouse culture of MP with anatomical integrity is possible from each individual part of the GIT and leads to the generation of neurospheres that can be used for further experiments such as transplantation or long time culture after differentiation. Moreover, the liberation of factors from the myenteric tissue is easily feasible because of the large amounts of tissue which can be used for the experiments. Due to the exceptional purity of the tissues, contaminating cell populations, such as smooth muscle cells, can be avoided. Thus, the data mirror the real situation within the ENS without destroying neural interactions between glial and neuronal populations.

The presented technique allows to perform various functional experiments, from the release of cytokines, the culture of MP with subsequent experiments, or calcium imaging and electrophysiological approaches, such as patch clamp or multi electrode array measurements.

Release experiments are easily to perform. We could measure low levels of IL-6 in the supernatant of free floating MP cultures from the small intestine of adult mouse after 72 hours in vitro. This is in line with published data that show that enteric glia cells can produce Il-6 in vitro^38^. Moreover, we found release of VEGF, a cytokine mainly known to induce angiogenesis. It is reported that IL-6 and VEGF levels correlate because IL-6 is able to induce the expression of VEGF in the central and peripheral nervous system. VEGF is produced by Schwann cells and astrocytes and exerts divergent functions including protecting neurons, acting as mitogens for Schwann cells and astrocytes, supporting the survival of definitive neural stem cells and increasing proliferation of neuronal progenitors in vitro^39^,^40^,^41^,^42^. This example shows that it is possible to identify soluble factors produced by the MP with this approach to investigate their specific functions on the ENS or the interaction of cells in health and disease in further experiments. This analysis can be performed with a certain spatial resolution that can deliver data with high precision and that is functionally related to distinct segments of the gut.

The proliferation of the cells from the individual segments was restricted to the glial cells similar to the homeostatic in vivo situation but depends on the culture conditions and the age of the mice used.

An additional aspect of the segmental isolation option is the use of "segmental-cultures" (esophagus, stomach, duodenum, jejunum, ileum or colon) for pharmacological testing. Here, the specific action of an individual drug can be evaluated concerning spatial preferences along the gut axis.

The presented technique provides a new perspective for the detailed spatiotemporal investigation of MP responses and alterations in health and disease.

In conclusion, the presented optimized and standardized approach provides a strong tool that enables anybody interested in ENS alterations to isolate and investigate MP from all ages and locations in adult human and mouse gut. This provides options for the detailed investigation of specific actions or reactions of the ENS along the gut axis. In mouse models, a spatiotemporal analysis of the changes within the ENS allows, e.g., the identification of specific protein changes in a certain segment, which might lead to translational consequences, such as taking biopsies from the stomach instead of the colon for the prediction of neurodegeneration because the changes might appear first in the stomach. In human tissue, influences of the disease on the ENS can be investigated on protein, genomic or cytokine levels with high purity. If applied to transgenic animal models and compared to the individual human disease, the proposed method will provide a new field of understanding and diagnoses for many diseases, including diseases not restricted to the gastrointestinal tract.

## Methods

All human samples were collected after written informed consent following the Helsinki declaration and with the approval of the local ethical committee of the Saarland (Ärztekammer). All experimental protocols were approved by the local ethical committee of Rhineland-Palatinate. All methods were carried out in "accordance" with the approved guidelines.

Human and animal samples were obtained from different states within Germany (border region), so that there were two distinct ethical approvals necessary.

### Animals

Heterozygous Nestin-GFP adult (≥10 weeks) or wild-type male and female mice were used for this study. The animals were raised and bred according to institutional guidelines.

### Human Tissue

Stomach tissue was collected from 4 patients undergoing surgical treatment for esophagus cancer after informed consent of the patients.

All human and animal studies have been approved by the local institutional review board.

### Isolation of the Myenteric Plexus

The GIT was dissected from mice using a binocular microscope. The mesenteries were completely removed, and the GIT was then divided in individual segments (stomach, duodenum, jejunum, ileum, caecum, proximal and distal colon) and was opened lengthwise. After a brief washing in MEM-Hepes (PAN) 1% Penicillin Streptomycin (Applichem) to remove the remaining luminal content, the muscle layer was dissected completely under a binocular microscope using watchmaker forceps (Dumont Nr.5) and was stored in MEM-Hepes PS on ice.

The muscle layers were cut into pieces of approximately 2 to 5 mm^2^ and placed in a 1.5-ml tube (Greiner Bio-One) containing neutral 830 μl HBSS (PAN) 1% penicillin streptomycin (Applichem), 150 μl Liberase TH (Roche) from stock solution and 20 μl DNAse (Roche). The tissue was allowed to digest for 4 h at 37°C and was then mechanically disrupted by a modest shaking to separate the MP networks with anatomical integrity from the surrounding smooth-muscle cells. The shaking procedure is critical and should be performed under visual control with a very low amplitude (less than 1 cm). The shaking should immediately be stopped as soon as the tissue structure of the smooth muscle disintegrates. The suspension containing the MP networks was placed in a 35-mm Petri dish (CELLSTAR) and fresh MEM-Hepes (PAN) 1% penicillin streptomycin (Applichem) was added to improve the visibility of the isolated MP-networks within the overall cell suspension. The supernatant containing mainly muscle cells was discarded when the MP networks were sedimented on the bottom of the Petri dish. This has to be performed under optical control to avoid the unintentional removal of already isolated plexus. The sedimented MP-networks were harvested with a pipette and transferred to fresh MEM-Hepes. If necessary, this last step was repeated twice to obtain a pure collection of MP without other contaminating tissues. The MP networks were centrifuged at 100 × *g* for 5 min and used for further experiments.

The isolation of the human MP was similar to that of the mouse MP, but the digestion had to be performed overnight in tissue culture medium. Images from the preparation were captured using a Zeiss Stemini 2000-C binocular connected with an Olympus E-330 camera.

### Propidiumiodid staining of myenteric plexus networks

In order to investigate the death rate of MP cells induced by isolation, propidium iodid staining was performed. MP networks were isolated as described above from nestin GFP mouse and transferred into a 35 mm petri dish containing 3 ml proliferation medium. Afterwards, 3 μl of 50 μg/ml propidium iodid stock solution (BD Pharmingen) was added and the MP networks were incubated 10 min at 37°C. The MP networks were collected and washed in a 35 mm petri dish with proliferation medium. The MP networks were collected again and transferred into a petri dish with a glass bottom (Greiner Bio-One). Image analysis was carried out with the CellObserver Z1 using Axiovision software.

### Cell Culture

For neurosphere generation, MP networks (organic culture) from stomach, duodenum, jejunum, ileum, caecum, proximal, as well as the distal part of the colon were incubated in 24-well plates (Greiner Bio-One) with proliferation medium (neurobasal medium (PAA), 1% BSA, Sigma-Aldrich; 1% penicillin streptomycin, Applichem; 0,25% glutamin, Sigma-Aldrich; 0,1% **ß**-mercaptoethanol, GIBCO; neuromix 2A without retinoic acid, PAA) containing 20 ng/ml bFGF and 10 ng/ml EGF (Immunotools). The medium was replaced on days 1 and 3. After 6 days, the neurospheres were collected and plated on ECM-coated coverslips (ECM, Sigma Aldrich, was diluted 1:200, plated on coverslips and incubated for 2 h at 37°C) and were incubated with differentiation medium (neurobasal medium (PAA), 1% BSA (Sigma-Aldrich), 1% penicillin streptomycin (Applichem), 0,25% Glutamine (Sigma-Aldrich), 0,1% ß-mercaptoethanol (GIBCO), Neuromix 2A with retinoic acid (PAA))) without any factors for 3–6 d. Then, the samples were fixed with 4% Formaldehyde for 1 h at RT. During the neurosphere culture, 5 μM BrdU was added.

### Immunofluorescence Staining of Differentiated Neurospheres

The cells were permeabilized with 0.5% Triton (10 min at RT) and blocked with 10% Normal Goat Serum (PAA, 1 h at RT) before incubating with primary antibodies (2 h at RT) against BrdU (rat, AbdSerotec) and S100b (rabbit, Sigma-Aldrich) or PGP (rabbit, Dako Cytomation). After three washings with PBS, primary antibodies were visualized using secondary antibodies (2 h at RT) anti-rat Alexa 350 and anti-rabbit Alexa 594 (Invitrogen). Finally, the coverslips were washed with distilled water and mounted on microscopic slides using mounting medium (Dako). Image analysis and processing was performed with the CellObserver Z1 using Axiovision software (Zeiss).

### Tissue processing for scanning electron microscopy

The MP networks were isolated as described above and fixed in 2% glutaraldehyde in 0.1 M cacodylate buffer. The following steps were carried out in 1.5-ml Eppendorf tubes. The MP networks were washed in cacodylate buffer (3 × 10 min). The tissue was incubated in 1% osmium (1 h) and rinsed in distilled water (1 h).

After incubation with 1% tannic acid (1 h), the tissue was repeatedly washed in distilled water (1 h). The tissue was dehydrated in a graded series of ethanol, starting at 70% for 30 min in each step. The ethanol was then replaced with hexamethyldilazane (HMDS) (ethanol/HMDS1:1 30 min, 2x HMDS 30 min). Finally, the Eppendorf tubes were opened for drying of the MP networks (overnight under a fume hood).

The dried networks were transferred on aluminum stubs and sputtered with carbon. Images were captured using a high resolution scanning electron microscope (Gemini, Zeiss).

### LPS treatment and 2D-Differential-in-gel-electrophoresis (DIGE)

Human MP was kept in tissue culture medium for 24 h with and without 5 μg/ml bacterial lipopolysachharide (Sigma). After this stimulation, the plexus pieces were collected, frozen in liquid nitrogen and then homogenized (FastPrep24, MP Biomedicals) in lysis buffer containing 7 M urea, 2 M thiourea, 4% CHAPS (Invitrogen, Germany), protease-inhibitor cocktail (Roche, Germany) and a nuclease-mix (GE-Healthcare, Germany). The proteins were purified using the 2-D clean-up kit (GE-Healthcare, Germany). Afterward, the protein pellets were re-equilibrated in buffer with 7 M urea, 2 M thiourea and 4% CHAPS and the amount of purified protein was analyzed (2-D quant-kit, GE-Healthcare, Germany).

Equal amounts of the two samples were labelled with different fluorescent dyes: Cy3 and Cy5. A mixture of both proteins was used as an internal standard and labelled with Cy2.

All three samples were then mixed, and a total amount of 55 μg of protein was applied to an IPG-Strip (Immobilized pH-Gradient) with an 18 cm, nonlinear gradient from pH 3–11 (GE) and focused for 8 h on the IPGphor3 (GE). The strips were transferred to a 12.5% polyacrylamide-gel and run on an EttanDalt12-separation unit overnight. After separation, the gels were scanned on a TyphoonTrio laser scanner (GE) and the spots were analyzed using the DeCyder (GE) software.

### Cytokine Measurement

The isolated MP from one small intestine of adult mouse was cultured in a culture flask as described above in 5 ml of proliferation medium in the presence of 20 ng/ml bFGF, 10 ng/ml EGF and 10 ng/ml GDNF. The supernatant was collected after 72 h and analyzed using multiplex assay (Luminex) for G-CSF, GMCSF, M-CSF, IL6, IL10, IL15, IL17, LIF, TNFα, Eotaxin and VEGF corresponding to the manufacturers' instructions (Millipore).

The results were displayed using GraphPad Prism software.

### MEA-analyses

In this study we used a commercial MEA Setup from Multi Channel Systems (MCS, Reutlingen, Germany) together with the provided software for data recording and analyses (MEA-Rack, Change-MEA, TCX-Control). This MEA-workstation (USB-MEA60-Inv-sytem) allowed us data recording and analyzing from up to 60 MEA electrodes. The amplifier platform was mounted on a shock isolated stereo optic microscope (Hundt, Germany) which was placed in a Faraday's cage. Inside the cage a gravity driven batch application system was installed.

Standard microelectrode arrays with an internal reference electrode were used. The electrode grid was 8 × 8 with 60 electrodes and an electrode spacing of 200 μm with an electrode diameter of 30 μm. An additional glass ring of 6 mm height and 28 mm diameter was mounted in the center of the MEA for cell culturing.

Enteric neurospheres from adult mouse gut were cultivated for one week before being placed into the center of the MEA-chip, that was previously coated with 5 mg/ml fibronectin. The intrinsic activity of short term cultured neurospheres was measured after 24 h, 72 h and 120 h on the chip. 15 to 19 days in vitro. Spike-analyses were done for 180 s in culture medium. For control and to exclude artifacts a cocktail of voltage-gated sodium channel blockers (NaV-blocker: 2 μM TTX, 100 μM zinc, and 100 μM copper) was applied at the end of each experiment after 120 h.

### Calcium imaging

Isolated MP networks were carefully resuspended in 5 μl collagen N gel (Amedrix, Esslingen, Germany). The MP network/collagen suspension was plated out as a thin layer on a 35 mm petri dish with a glass bottom (Greiner Bio-One) in order to fix the MP networks in one plane. The MP networks were carefully dispersed with forceps if necessary. The samples were incubated 30 min at 37°C for polymerization of the collagen N gel. Then the networks were washed three times with recording buffer. The recording buffer was composed of 150mM NaCl (Applichem), 5mM KCl (Roth), 2mM CaCl_2_ (Applichem), 1mM MgCl_2_ (Applichem), 10mM Hepes (Sigma Aldrich) and 1,5 g/LGlucose (Applichem). The staining solution consisting of recording buffer, 200 μg/ml Pluronic (Sigma Aldrich) dissolved in DMSO (Applichem), 2 μg/ml Fluo-8 (AAT Bioquest) dissolved in DMSO and 0,5% FCS (PAN) was added for 30 min at RT protected from light. The MP networks were again rinsed three times in recording buffer and used for the measurements. Potassium chloride was used for depolarization. The measurement was carried out with the CellObserver Z1 using the Physiology module of the Axiovision software (Zeiss).

## Supplementary Material

Supplementary Information Supplementary Information
