# Supplementary material for: Isolation of high-purity myenteric plexus from adult human and mouse gastrointestinal tract
Source: Sci Rep. 2015 Mar 20;5:9226. doi: 10.1038/srep09226 (PMC4366762; doi:10.1038/srep09226)
Supplement: Supplementary Information [file srep09226-s1.docx]

**Isolation of high-purity myenteric plexus from adult human and mouse gastrointestinal tract**

David Grundmann^1*^, Markus Klotz^1^, Holger Rabe^1^, Matthias Glanemann^2^, Karl-Herbert Schäfer^1*^

Supplementary material


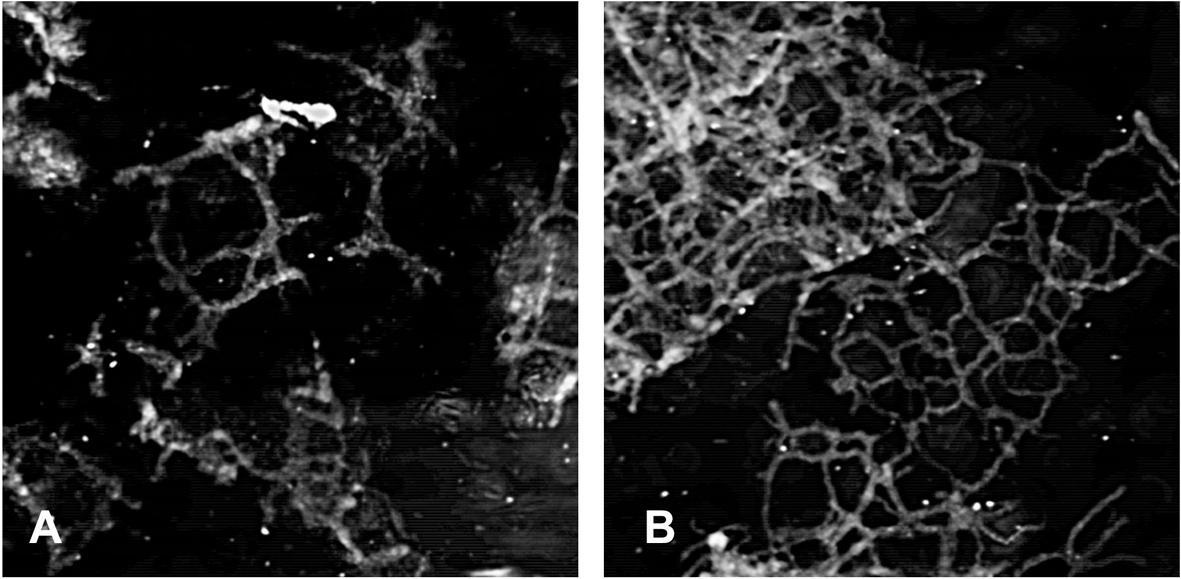


Suppl. Figure 1:

Isolated myenteric tissue after collagenase, respectively Liberase digestion. When Collagenase II was used for digestion only single fragments of myenteric networks could be harvested in case high concentrations of CLSII were used (A). In contrast Liberase digestion delivers high quantities of completely isolated and intact myenteric networks of high purity (B).


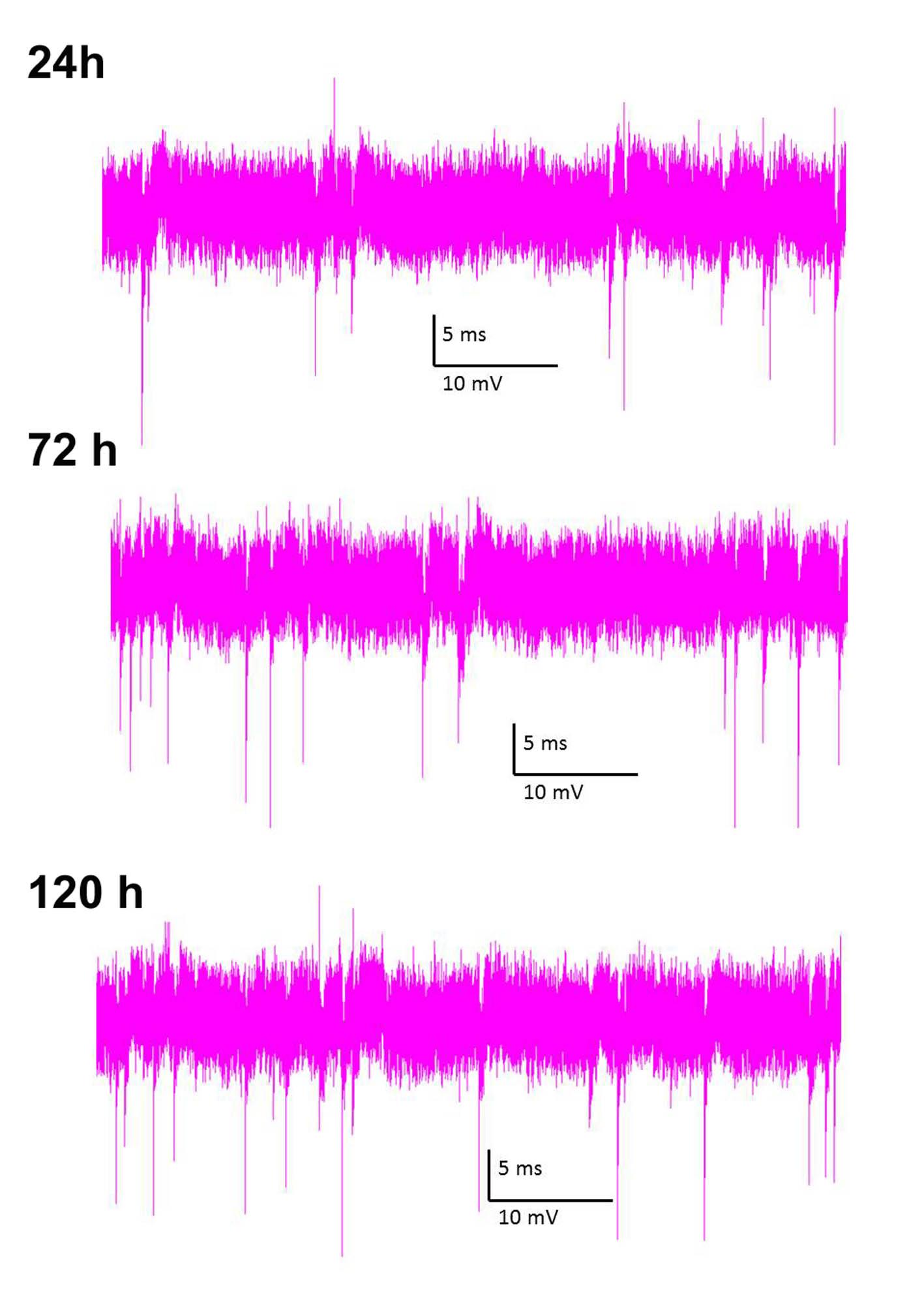


Suppl. Figure 2:

Intrinsic spike activity of ENS-neurospheres plated out on MEA-chips in culture media. MEA-recording of neurospheres was done after 24 h, 72 h, and 120 h on the chip. The spike frequency increased strongly from 24 h to 72 h, whereas after 120 h no further increase in spiking activity was visible.


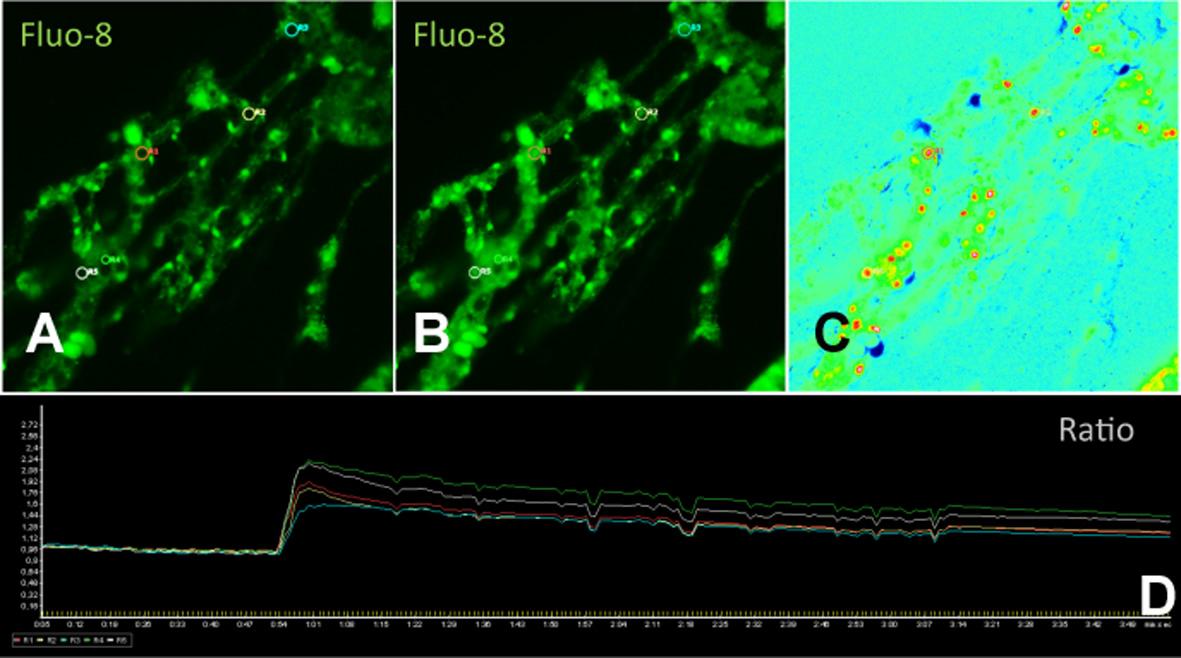


Suppl. Fig.3:

Calcium imaging of myenteric plexus network from ileum of adult mouse. Myenteric networks from ileum of adult mouse were attached on the glass bottom of a petri dish using collagen N. Cells were loaded with Fluo-8 (A) and depolarized (B) using potassium chloride. Calcium signals could be observed and analyzed in several myenteric cells (C, D).
